# Prevalence of carrier state theileriosis in lactating cows

**DOI:** 10.14202/vetworld.2017.1471-1474

**Published:** 2017-12-14

**Authors:** Niranjana Sahoo, Bikash Kumar Behera, Hemant Kumar Khuntia, Manojita Dash

**Affiliations:** 1 Department of Epidemiology & Preventive Medicine, College of Veterinary Science & Animal Husbandry, Orissa University of Agriculture and Technology, Bhubaneswar, 751003, Odisha, India; 2 Centre for Wildlife Health, College of Veterinary Science & Animal Husbandry, Orissa University of Agriculture and Technology, Bhubaneswar, 751003, Odisha, India; 3 ICMR-Regional Medical Research Centre, Department of Health Research, Ministry of Health & Family Welfare, Government of India, Bhubaneswar, 751023, Odisha, India

**Keywords:** bovine theileriosis, carrier state, polymerase chain reaction, *Theileria annulata*, *Theileria orientalis*

## Abstract

**Aim:**

The objective of this study was to examine the carrier status of theileriosis among apparently healthy cross-bred jersey cattle population of Odisha using conventional blood smear examination and polymerase chain reaction (PCR).

**Materials and Methods:**

A total of 34 apparently healthy cross-bred Jersey lactating cows were considered in this study. Blood samples were subjected to microscopic examination after staining with Giemsa stain and PCR based molecular diagnosis using two sets of primer, i.e., N516/N517 and TorF1/TorF2 specific for *Theileria annulata* and *Theileria* *orientalis*, respectively.

**Results:**

Examination of blood samples revealed presence of theileria parasites to a magnitude of 20.59% for *T. annulata*, 8.82% for *T*. *orientalis*, and 2.94% for both.

**Conclusion:**

Molecular diagnosis was found to be much more sensitive than conventional method for diagnosis of theileriosis. *T. annulata* was found to be the predominant species affecting the exotic cattle. *T. orientalis* was detected in apparently healthy cows.

## Introduction

Hemoprotozoan diseases in general and theileriosis in particular are considered as a serious economic threat for dairy farmers due to its adverse effects in the form of mortality and low productivity particularly in the tropical and subtropical regions of the world [1].

Continuous change in climatic conditions in the past few decades resulting in high environmental temperature and humidity has favored tick multiplication and thereby a gradual surge in the incidence of tick-borne menace [2]. Increased population and introduction of the exotic/crossbred cattle population, especially in the endemic areas, has magnified susceptibility to theileriosis. Predisposing factors linked with stress such as high production, low nutrition, poor housing, unhygienic indoor condition, and development of drug resistance to acaricidal agents have further intensified the adverse impact of theileriosis.

India has long been established as a victim of theileriosis. A review of the literature on the incidence of theileriosis in India over the past four decades has revealed its existence in most states of India [3], including Odisha [4,5]. Most of the research work in India are focused on bovine tropical theileriosis caused by *Theileria annulata*. However, during the past few years, reports on *Theileria orientalis* associated clinical infections have been accumulated from different states of India such as Tamil Nadu, Kerala, and Assam [6-9]. Thus, identification of species-specific theileriosis is essentially required to unveil the picture of bovine theileriosis in the areas of interest. Besides, differential diagnosis of the diseases having clinical findings similar to each other is of utmost importance to avoid polypharmacy especially in high yielders having enhanced vulnerability to theileriosis [10].

Clinical signs such as fever, inappetence, anemia, coughing, dyspnea, reduced milk yield, swollen subcutaneous lymph nodes, abortion, icterus, hemoglobinuria and soil licking, raise suspicion of clinical theileriosis, and pave way for confirmatory diagnosis whereas the animals with low infectivity remain undiagnosed and continue to be a constant source of infection for other susceptible hosts. Keeping the above facts in forefront, the present study was conducted to diagnose occult or carrier status, if any, by microscopy and polymerase chain reaction among crossbred lactating cows in an area of Odisha, India, reported to be endemic for theileriosis.

## Materials and Methods

### Ethical approval

Laboratory tests required for the investigation were in accordance to the guidelines provided in the Institutional Animal Ethics Committee, and were performed in the Department of Veterinary Epidemiology and Preventive Medicine. The study period extended from March 2016 to February 2017.

### Experimental design

Blood samples were collected from apparently healthy crossbred Jersey lactating cows reared in small private farms (head size: 2-8) of Niali block of Cuttack district of Odisha bearing GPS coordinates 20.1411° N and 86.0606° E. Cows selected in the study were between 3^rd^ and 6^th^ lactation, within 2 months of parturition and with yielding record of minimum 10.0 L per day. A total of 34 cows were sampled in the study. 1 mL blood sample from each cow was collected properly from jugular vein in EDTA coated vacutainer tubes during morning hours between 5.00 and 7.00 am. Thin blood smears were prepared from the freshly collected blood samples, and the rest was stored at 4°C for subsequent use in PCR.

### Microscopic examination

Thin blood smear was prepared taking one drop (50 µl) of blood on a clean glass slide and spreading it with another spreader slide at an angle of 45°. Prepared smear was fixed with methanol for 5 min followed by flooding with 10% Giemsa’s solution for 40 min. Blood smears were carefully examined for *Theileria* parasites under the oil immersion lens (100× magnifications). Presence of piroplasm(s) inside the erythrocytes was considered positive for *Theileria* spp. infection.

### DNA extraction and PCR

Genomic DNA was extracted from each blood sample using commercially available DNA mini kit (QIAGEN, USA). According to the manufacturer‘s instructions, 200 μl of whole blood was used for each sample. The concentration of extracted DNA was checked by agarose gel electrophoresis. This purified DNA was used as a template for the PCR. Two sets of primers were used in the investigation, i.e., N516/N517 (primer set A) for *T. annulata* and TorF1/TorF2 (primer set B) for *T. orientalis* (Table-1). PCR was performed in a final reaction volume of 25 µl reaction mixtures containing 2 µl DNA sample (using 30 ng/µl template in case of DNA reference samples), 50 mM KCl, 10 mM Tris–HCl (pH 8.3), 1.5 mM MgCl_2_, 200 µM of dNTP mix, 20 pmol of each primer, and 0.5U Taq polymerase enzyme and sterile distilled water up to 25 µl. The reaction mixture was placed on a heating block of a programmable thermocycler (Life technologies, Thermo Fischer scientific). After a denaturation step of 5 min at 94°C, each of 30 cycle consisted of 1 min at 94°C, 1 min at 55°C, 1 min at 72°C followed by 10 min at 72°C for *T. annulata* whereas for *T. orientalis* after denaturation of 5 min at 94°C, each of the 35 cycles consisted of 1 min at 94°C, 30 s at 55°C, 45 s at 72°C, and final extension of 5 min at 72°C. Positive control and negative control samples were run along with the test samples. The amplification products were subjected to electrophoresis on 1.5% agarose gel with a ladder and the amplified products were visualized using gel documentation system.

**Table-1:** Primer sets used for PCR.

Oligo name	Sequence (5’→3’)	Product size	Target genome	References
N516	GTAACCTTTAAAAACGT	721 bp	*T. annulata* specific	[21]
N517	GTTACGAACATGGGTTT			
Tor F1	CTTTGCCTAGGATACTTCCT	776 bp	*T. orientalis* specific	[22]
Tor R1	ACGGCAAGTGGTGAGAACT			

PCR=Polymerase chain reaction, *T. annulata=Theileria annulata*, *T. orientalis=Theileria orientalis*

Amplified products were randomly selected and sent to Bhat bio-tech India private limited, Bangalore, Karnataka, for sequencing.

## Results

Morphologically, under microscopic examination of blood smears positive for *Theileria* spp. infections, intraerythrocytic bodies appeared in the form of dot shape in *T. annulata* and slightly elongate in case of *T. orientalis* [11]. Examination of 34 Giemsa stained blood smears from apparently healthy cows, 3 (8.82%) samples showed the presence of *Theileria* spp. parasite. Schizonts could not be demonstrated in any of these blood smears.

**Figure-1:**
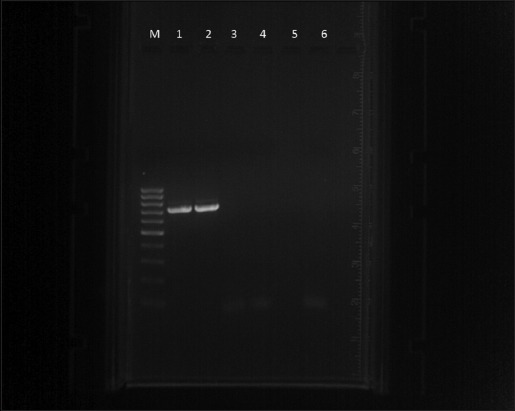
Agarose gel electrophoresis of polymerase chain reaction products: Lane M – 100 bp Marker, Lane 1 – positive control for *Theileria annulata*, Lane 2 – positive sample, Lane 3-5 – Negative samples, and Lane 6 – Negative control.

**Figure-2:**
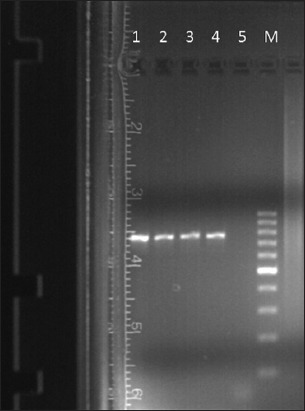
Agarose gel electrophoresis of polymerase chain reaction products: Lane 1 – positive control for *Theileria* *orientalis*, Lane 2-4 – positive samples, Lane 5 – Negative control and Lane M – Marker DNA (100 bp).

Further examination of the blood samples on PCR showed a different picture. PCR reactions showing a product size of 721 bp by primers N516/N517 and a product size of 776 bp by primers TorF1/TorR1 were considered positive for *T. annulata* (Figure-1) and *T. orientalis* (Figure-2), respectively. Of all the 34 blood samples of the apparently healthy lactating cows, the presence of *T*. *orientalis* and *T. annulata* was found in 7 (20.59%) and 3 (8.82%) cows, respectively, while both the species were detected in a solitary case (2.94%). These findings established the carrier stage of *T. annulata* and *T*. *orientalis*, either single and/or mixed form, in 11 (32.35%) crossbred Jersey Milch cows.

Sequencing of the amplified PCR products confirmed that the product has 100% sequence homology with *T. annulata* and *T*. *orientalis*.

## Discussion

Odisha is situated along the eastern coast of India between 18°2′ and 22°6′ N latitude and 82°8′ and 87°6′ E longitude. The state experiences moderately hot and very humid climate favoring rapid multiplication of vectors and thereby propagation of vector-borne diseases in the state. Of such diseases, bovine theileriosis is one of the most destructive obstacles to livestock production with massive economic repercussions [12]. At least five species of *Theileria* spp. (*T. annulata, Theileria parva, Theileria taurotragi, Theileria velifera* and members of *Theileria sergenti/orientalis/buffalo* group) have been found to infect cattle. The present study unveiled the carrier status of *T. annulata* and *T. orientalis* in Odisha, a coastal state in eastern part of India.

Although the conventional Giemsa stained blood smear examination considered to be the gold standard method for identification of intraerythrocytic and Schizont stage of *Theileria* spp. parasite, this method is rarely successful in case of carrier animals [13]. This is in line with the findings in the present study where very low (8.82%) proportion of positivity was recorded as against 32.35% in molecular tests. Moreover, by microscopic examination, it is difficult to discriminate different species of *Theileria* spp. that may occur either as single or mixed form within the same bovine host [14]. The serological tests are also not suitable due to cross-reactivity with other *Theileria* spp. [15] and inability to distinguish between active carriers and animals with antibodies due to prior infections [16]. Limited information is available regarding the role played by these carrier animals with respect to their ability to transmit the disease since they usually remain undetected being asymptomatic [17]. It is reported that most assays may detect >400,000 parasites/L blood and natural parasitaemia distribution in carrier state animals seems to be above this limit of detection, suggesting that most molecular assays should be able to detect the majority of infected individuals under endemic conditions. To overcome these constraints, PCR was preferred for its high sensitivity and the ability to amplify even a minute concentration of parasitic DNA in the blood enabling the detection of carrier animals even in endemic conditions [18].

Using PCR, *T. orientalis* was found to be the predominant species prevailing in the state of Odisha. Besides *T. annulata*, the prevalence of *T. orientalis* infections was seen for the first time in Odisha which has earlier been documented in Tamil Nadu [7] and Assam [11]. Mixed infection was found in one case which has previously been reported in Tamil Nadu [8].

Appearance of carrier animals in endemic areas is a matter of concern. Carrier state is usually chronic in nature [2]. Such category of infected animals with the tick feeding on them might be the source of infection when reared together with healthy cattle. Moreover, disease outbreak due to *T. orientalis* may represent a hidden burden to livestock productivity in regions where infection is endemic [19]. *T. orientalis* has also been found capable of being mechanically transmitted to healthy cattle by minute volumes of blood through intravenous inoculation as well as through biting arthropods [20]. Hence, it is not unwise to infer that this mode of transmission, not unlikely in field conditions, might be contributing a lot in dissemination of the disease through multiple use of unsterilized needles during the therapeutic process. The present PCR based assay helped to throw light on carrier stage of the *Theileria* spp. both *T. annulata* and *T*. *orientalis* in Odisha. The results of the study would be of immense help in proper planning and effective control of theileria infection.

## Conclusion

Examination of blood samples from 34 apparently healthy cross-bred Jersey lactating cows in an endemic area exposed the presence of *Theileria* spp. in 3 (8.82%) on blood staining and through PCR in 11 (32.35%) cows, thereby confirmed its carrier status, either single or mixed form. *T. orientalis* constituted the major share as compared to *T. annulata*.

## Authors’ Contributions

MD collected the samples from the field and carried out the preliminary laboratory work. BKB performed the molecular work of the study under the guidance of HKK. NS supervised the entire laboratory activities. NS and HKK prepared manuscript whereas former revised the same. All authors read and approved the final manuscript.
